# Comparing diagnostic and prognostic performance of two-gene promoter methylation panels in tissue biopsies and urines of prostate cancer patients

**DOI:** 10.1186/s13148-018-0564-2

**Published:** 2018-10-29

**Authors:** Catarina Moreira-Barbosa, Daniela Barros-Silva, Pedro Costa-Pinheiro, Jorge Torres-Ferreira, Vera Constâncio, Rui Freitas, Jorge Oliveira, Luís Antunes, Rui Henrique, Carmen Jerónimo

**Affiliations:** 1 Cancer Biology and Epigenetics Group, IPO Porto Research Center (CI-IPOP), Portuguese Oncology Institute of Porto (IPO Porto), Porto, Portugal; 2 Department of Pathology, Portuguese Oncology Institute of Porto (IPO Porto), Rua Dr. António Bernardino de Almeida, 4200-072 Porto, Portugal; 3 Department of Urology, Portuguese Oncology Institute of Porto (IPO Porto), Rua Dr. António Bernardino de Almeida, 4200-072 Porto, Portugal; 4 Department of Epidemiology, Portuguese Oncology Institute of Porto (IPO Porto), Rua Dr. António Bernardino de Almeida, 4200-072 Porto, Portugal; 5 Department of Pathology and Molecular Immunology, Institute of Biomedical Sciences Abel Salazar (ICBAS), University of Porto, Porto, Portugal; 6 Cancer Biology and Epigenetics Group - Research Center (LAB3), Portuguese Oncology Institute of Porto, Rua Dr. António Bernardino Almeida, 4200-072 Porto, Portugal

**Keywords:** Prostate cancer, DNA methylation, Biomarkers, Methylation test, Detection, Prognosis

## Abstract

**Background:**

Prostate cancer (PCa) is one of the most common cancers among men worldwide. Current screening methods for PCa display limited sensitivity and specificity, not stratifying for disease aggressiveness. Hence, development and validation of new molecular markers is needed. Aberrant gene promoter methylation is common in PCa and has shown promise as clinical biomarker. Herein, we assessed and compared the diagnostic and prognostic performance of two-gene panel promoter methylation in the same sample sets.

**Methods:**

Promoter methylation of panel #1 (singleplex-*miR-34b/c* and *miR-193b*) and panel #2 (multiplex*-APC*, *GSTP1*, and *RARβ2*) was evaluated using MethyLight methodology in two different cohorts [prostate biopsy (#1) and urine sediment (#2)]. Biomarkers’ diagnostic (validity estimates) and prognostic (disease-specific survival, disease-free survival, and progression-free survival) performance was assessed.

**Results:**

Promoter methylation levels of both panels showed the highest levels in PCa samples in both cohorts. In tissue samples, methylation panel #1 and panel #2 detected PCa with AUC of 0.9775 and 1.0, respectively, whereas in urine samples, panel #2 demonstrated superior performance although a combination of *miR-34b/c*, *miR-193b*, *APC*, and *RARβ2* disclosed the best results (AUC = 0.9817). Furthermore, higher *mir-34b/c* and panel #2 methylation independently predicted for shorter DSS. Furthermore, time-dependent ROC curves showed that both *miR-34b/c* and *GSTP1* methylation levels identify with impressive performance patients that relapse up to 15 years after diagnosis (AUC = 0.751 and AUC = 0.765, respectively).

**Conclusions:**

We concluded that quantitative gene panel promoter methylation might be a clinically useful tool for PCa non-invasive detection and risk stratification for disease aggressiveness in both tissue biopsies and urines.

**Electronic supplementary material:**

The online version of this article (10.1186/s13148-018-0564-2) contains supplementary material, which is available to authorized users.

## Background

Prostate cancer (PCa) remains a major public health concern in male gender mainly due to increased life expectancy and population aging [1]. This neoplasm is usually clinically silent until extra-prostatic invasion or metastatic occurs, entailing the need for development and implementation of effective screening methods that allow for detection of PCa while still confined to the prostate, at a potentially curable stage. Moreover, PCa is a quite heterogeneous disease that ranges from clinical indolent to aggressive behavior and patients’ risk stratification remains a clinical challenge [2, 3]. Notwithstanding recent advances in characterization of PCa biology, significant challenges concerning adequate PCa management remain, as new stratification methods and its clinical validation are still needed [4].

Aberrant DNA methylation, the most widely studied epigenetic mechanism in human cancers, is a very prevalent and specific feature of PCa [5]. Furthermore, altered DNA methylation patterns represent early events during prostate carcinogenesis and constitute the most frequent epigenetic phenomenon in both localized and metastatic PCa [6, 7]. Importantly, DNA methylation alterations are sufficiently stable and easily quantifiable in liquid biopsies [8, 9], which constitute a valuable and minimally invasive means of interrogating the presence of tumor cell DNA in bodily fluids.

Previous studies have identified several epigenetic-based biomarkers with great potential for detection of clinically relevant PCa [revised in [5]], including the ProCaM assay (which evaluates *GSTP1*, *APC*, and *RARβ2* promoter methylation in urine samples), as a positive result correlates with increased likelihood of finding high-grade PCa in prostate biopsy [10]. More recently, we demonstrated that quantitative *miR-193b* and *miR-34b/c* promoter methylation might be useful for non-invasive detection/diagnosis and prognostication, both in tissue and urine samples [11]. Indeed, besides representing promising PCa detection/diagnostic biomarkers, several studies demonstrated that gene promoter hypermethylation might add relevant prognostic information, such as *GSTP1* promoter methylation which independently predicted recurrence after radical prostatectomy [12] and higher *APC* promoter methylation levels which also showed independent prognostic value in addition to tumor stage [13]. Thus, DNA methylation-based biomarkers may add value in clinical practice as ancillary tests to assist in therapeutic decision-making.

## Methods

### Aim

Hence, the purpose of this study was to compare the diagnostic and prognostic value of two previously reported panels of methylated gene promoters, panel #1 (non-coding protein genes: *miR-193b* and *miR-34b/c*) [11] and panel #2 (protein coding genes: *APC*, *GSTP1*, and *RARβ2*) methylation levels [10] in the same series of samples, aiming at the establishment of an assay that may be further clinically validated.

### Patients and sample collection

Two independent case control retrospective cohorts of PCa patients were included in this study. The first comprises 74 patients with elevated PSA levels submitted to prostate biopsy from 2001 to 2003 at Portuguese Oncology Institute of Porto (IPO Porto, cohort #1). For each case, in addition to the standard diagnostic biopsy cores, one tissue core sample was collected from the more suspicious area, frozen at − 80 °C and subsequently cut in a cryostat for DNA extraction. For each case, a 4-μm section was cut, stained, and examined by an experienced pathologist, to confirm the presence of malignant cells and grading. As control samples, 16 morphologically normal prostate tissues (MNPT) were collected from bladder cancer patients, without concomitant PCa, submitted to cystoprostatectomy. All specimens were immediately collected after surgical procedures and frozen at − 80 °C and histologically confirmed for the absence of any tumor foci. Additionally, a second cohort was composed of 87 PCa patients, primarily diagnosed from 1999 to 2002 at IPO Porto, which voluntarily provided early morning voided urine samples (20–30 mL) before radical prostatectomy without previous prostatic massage (cohort #2). For control purposes, urine samples were collected from 32 asymptomatic donors at IPO Porto (2009 to 2010). After collection, urine samples were centrifuged at 4000 rpm for 20 min at 4 °C, and the obtained pellets were washed in PBS 1× and frozen at − 80 °C.

Relevant clinical data was retrieved from clinical records and are shown in Tables 1 and 2. Concerning assessment of prognosis, disease-specific survival (DSS, i.e., time between diagnosis and death or last follow-up) and disease-free survival (DFS, i.e., time calculated using the interval between date of curative treatment and date of biochemical relapse or date of last follow-up or date of death, if relapse was not observed) were computed. Biochemical relapse was considered when patients presented two consecutive risings of serum PSA levels ≥ 0.2 ng/mL after surgery or 2 ng/mL above the PSA nadir after radiotherapy. Progression-free survival (PFS) was calculated from the date of androgen deprivation therapy to the date of biochemical progression, date of last follow-up, or death, if due to other causes but PCa.

**Table 1:** Clinical and pathological data of morphologically normal prostatic tissue and prostate cancer patients submitted to a prostate biopsy (cohort #1) included in this study

	(Cohort #1)	
Clinicopathological data	MNPT	PCa (biopsies)
Patients, *n*	15	74
Median age, years (range)	64 (45–80)	69 (51–85)
Median PSA (ng/mL) (range)	n.a.	18.22 (4.52–542.00)
Clinical stage, *n* (%)		
II	n.a.	48 (64.86)
III	n.a.	12 (16.22)
IV	n.a.	14 (18.92)
Prognostic grade group, *n* (%)		
1	n.a.	30 (40.54)
2	n.a.	17 (22.97)
3	n.a.	16 (21.62)
4	n.a.	7 (9.46)
5	n.a.	4 (5.41)
CAPRA score, *n* (%)		
Low risk (0–2)	n.a.	7 (9.46)
Intermediate risk (3–5)	n.a.	26 (35.14)
High risk (6–10)	n.a.	41 (55.41)
D’Amico risk classification, *n* (%)		
Low risk		7 (9.46)
Intermediate risk		23 (31.08)
High risk		44 (59.46)
Treatment		
Radical prostatectomy/radiotherapy		39 (52.70)
Hormonotherapy		35 (47.30)
Follow-up		
Median (months, IQR)	n.a.	104.04 (67.03–145.48)
Patients without remission, *n* (%)	n.a.	3 (4.05)
Biochemical recurrence, *n* (%)	n.a.	13 (33.33)
Progression of disease, *n* (%)	n.a	16 (45.71)
Death due to PCa, *n* (%)	n.a.	13 (17.57)

*MNTP* morphologically normal prostate tissue, *PCa* prostate cancer, *IQR* interquartile range, *n.a* not applicable

**Table 2:** Clinical and pathological features of urine samples from asymptomatic controls and prostate cancer patients enrolled in this study (cohort #2)

	Urine (cohort #2)	
Clinicopathological data	AC	PCa
Patients, *n*	32	87
Median age, years (range)	58 (50–64)	64 (47–75)
Median PSA (ng/mL) (range)	n.a.	8.80 (3.50–20.40)
Pathological stage, *n* (%)		
pT2	n.a.	43 (49.43)
pT3a	n.a.	35 (40.23)
pT3b	n.a.	9 (10.34)
Prognostic grade group, *n* (%)		
1	n.a.	34 (39.08)
2	n.a.	39 (44.83)
3	n.a.	7 (8.05)
4	n.a.	5 (5.75)
5	n.a.	2 (2.30)

*AC* asymptomatic controls, *PCa* prostate cancer, *n.a* not applicable

### DNA extraction and sodium bisulfite treatment

DNA was extracted from clinical samples using phenol-chloroform method as described elsewhere [13]. Moreover, genomic DNA extracted from each clinical sample was submitted to bisulfite sodium conversion using EZ DNA Methylation-Gold™ Kit (Zymo Research, CA, USA) according to the manufacturer’s recommendation.

### Methylation analysis

The promoter methylation status of panel #1 (*miR-193b* and *miR-34b/c*) was determined as previously described in [11]. Briefly, methylation assessment of panel #1 was performed by quantitative methylation using KAPA SYBR FAST qPCR Kit (Kapa Biosystems, MA, USA). Primer sequences (Additional file 1: Table S1) were designed using Methyl Primer Express 1.0 and purchased from Sigma-Aldrich (MO, USA).

Panel #2 (*APC*, *GSTP1*, and *RARβ2*) methylation levels were evaluated using multiplex MethyLight methodology. The multiplex MethyLight assay was carried out in a reaction volume of 10 μL in 96-well plates using a 7500 Sequence Detector. The scorpion primer-probe sequences were the previously published in [14, 15], except for *APC* (Additional file 1: Table S2). Briefly, per each well, 5 μL KiCqStart™ Probe qPCR ReadyMix™ (Low ROX) (Sigma-Aldrich, Germany), 300 nM of each primer inner (Sigma-Aldrich, Germany); 100 nM of scorpion primer-probe for *APC*, *RARβ2,* and *β-Actin*, and 150 nM of scorpion primer-probe for *GSTP1* (Sigma-Aldrich, Germany) and 3 μL of bisulfite modified DNA as a template were added. The PCR program consisted of 95 °C for 5 min and 40 cycles at 95 °C for 15 s, and 64 °C for 1 min and 72 °C for 10 s. All samples were run in triplicate, and *β-Actin*, a housekeeping gene, was used as reference gene to normalize the results obtained for each gene studied.

### Statistical analysis

Differences in methylation levels and relationships between methylation and different clinical variables were assessed using the Kruskal-Wallis and Mann-Whitney non-parametric tests in multiple groups (more than two) and pairwise comparisons, respectively. *P* values were considered statistically significant if inferior to 0.05 for comparisons between two groups. In multiple comparisons and when statistically significant, Bonferroni’s correction was applied for pairwise comparisons, dividing the original *P* value by the number of groups. Spearman non-parametric correlation test was performed to test for associations between methylation levels and patient’s age and serum PSA.

For each gene promotor, receiver operator characteristics (ROC) curves were constructed by plotting the true positive (sensitivity) against the false-positive (1-specificity) rate, and area under the curve (AUC) was calculated. For the two panels, ROC curves were constructed using logistic regression model, to assess whether biomarker performance was increased using the panel. Specificity, sensitivity, positive predictive value (PPV), negative predictive value (NPV), and accuracy were determined for the gene-panel considering positive for the test when at least one of the genes was plotted as positive in individual analysis. The positive (LR+) and negative (LR-) likelihood ratios were also determined, and as the quantitative value of a calculated likelihood ratio is further away from 1 in either direction (> 1 for LR+ and < 1 for LR−), there is increasing utility of a diagnostic test to point toward, or away from, a diagnosis which indicate the value of performing the respective diagnostic tests. For this, the empirical cutoff obtained by ROC curve analysis [sensitivity + (1-specificity)] was established for each gene. This cutoff value combines the maximum sensitivity and specificity, ensuring perfect categorization of the samples as positive and negative for methylation test. In addition, time-dependent ROC curves were constructed considering biochemical recurrence/progression of disease for all tested genes at three-time points (5, 10, and 15 years) as endpoint.

DSS, DFS, and PFS curves (Kaplan-Meier with log rank test) were constructed considering clinicopathological variables (PSA levels, histologic grade group according with Epstein classification [16], clinical stage, CAPRA score [17], D’Amico’s risk group classification system [18]) and categorized promoter methylation status (using percentile 75 as cutoff). A Cox-regression model (multivariable model) was computed considering all significant clinical variables, to assess the relative contribution of each variable to the follow-up status. For multivariable testing, prognostic grade group (GG), clinical stage, PSA serum levels, CAPRA Score, and D’Amico’s classification were coded into two groups each [GG1 vs. GG 2–5, T2 vs. T3–4, PSA < 10 ng/mL vs. PSA ≥ 10 < 20 ng/mL vs. PSA ≥20 ng/mL, CAPRA Score low and intermediate risk (0–5) vs. high risk (6–10) [19], D’Amico’s classification low and intermediate risk vs. high risk].

Statistical analysis was carried out using SPSS Statistics, version 25 (IBM-SPSS, IL, USA), GraphPad Prism 7.01 (GraphPad Software, CA, USA) and R software version 3.2.5.

## Results

### Diagnostic performance of genes’ promoter methylation in prostate biopsy (cohort #1) and urine sediments (cohort #2) using different panels

To determine the performance of the gene methylation panels as PCa detection tools, ROC curve analysis was carried out and an empirical cutoff value was defined for calculation of biomarker performance (Additional file 1: Tables S3 and S4).

**Fig. 1:**
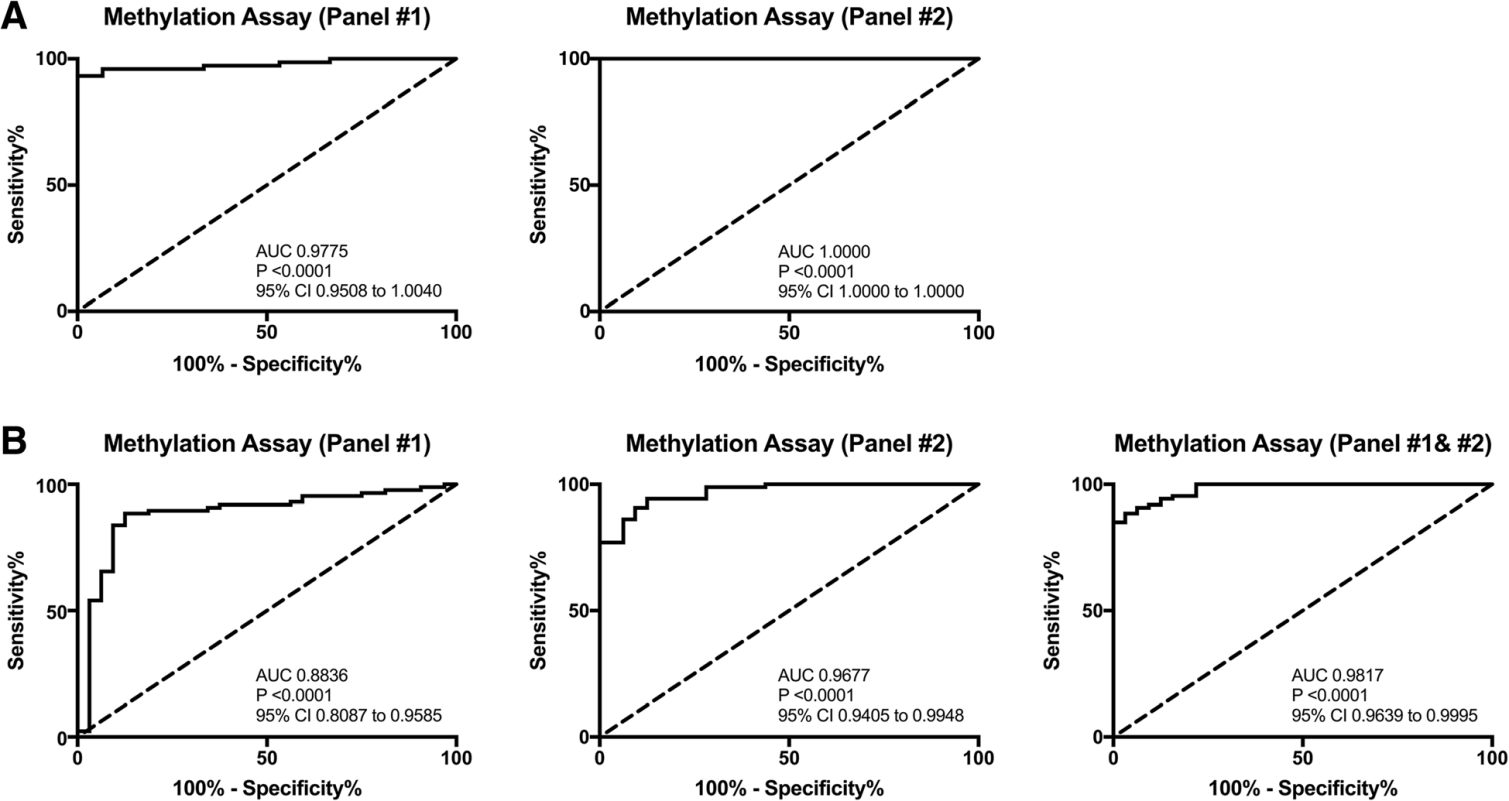
**a** Receiver operation characteristic (ROC) curves methylation-panel # 1 (*miR-34b/c*, *miR-193b*) and #2 (*APC*, *GSTP1*, and *RARβ2*) in cohort #1. (Reference line is the dashed line and ROC curve methylation panel is the solid line). **b** Receiver operation characteristic (ROC) curves methylation-panel # 1 (*miR-34b/c*, *miR-193b*), #2 (*APC*, *GSTP1*, and *RARβ2*) and combination of two methylation panels #1 and #2 in cohort #2

In prostate biopsy (cohort #1), methylation levels of all gene promoters were significantly higher in PCa patients compared to controls (Additional file 2: Figure S1). Moreover, panel #1 (*miR-193b* and *miR-34b/c*) discriminated PCa from non-cancerous prostate tissue with 97.3% sensitivity and 80.0% specificity, whereas panel #2 (*APC*, *GSTP1*, and *RARβ2*) displayed maximal sensitivity and specificity (Table 3 and Fig. 1a).

In urine sediments (cohort #2), all tested genes, except for *GSTP1*, displayed significantly higher promoter methylation levels in samples from PCa patients comparing with controls (Additional file 3 Figure S2). Panel #1 disclosed the highest sensitivity (95.4%), providing 92.4% accuracy and an AUC of 0.8836 (Table 4 and Fig. 1b). Panel #2 also displayed high sensitivity and specificity, although with an inferior positive predictive value (84.4%) (Table 4 and Fig. 1b). Interestingly, when both panels were combined, PCa was detected in voided urine with 100% sensitivity and an AUC value of 0.9817 (Table 4 and Fig. 1b).

### Association between promoter methylation levels and clinicopathological parameters

**Fig. 2:**
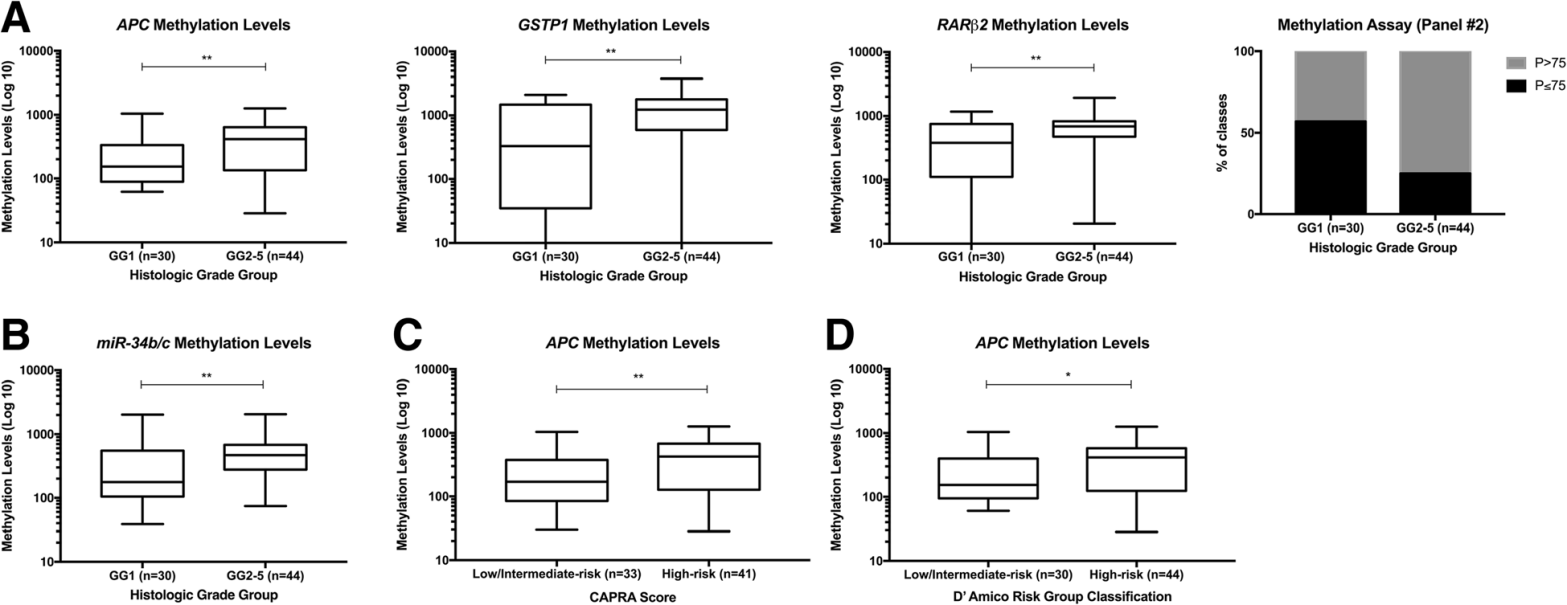
Promoter methylation levels of panel #2 (*APC*, *GSTP1*, and * RARβ2*) and *miR-34b/c* according to histologic grade group (**a**, **b**), respectively; Promoter methylation levels of *APC* according to CAPRA Score categories (**c**) and *APC* according to D’Amico risk group classification (**d**) in prostate biopsy tissue samples (cohort #1). (Mann-Whitney *U* test, **P < 0.01, *P < 0.05)

In cohort #1, higher methylation levels of panel #2 gene promoters significantly associated with higher prognostic grade group (GG2–5 vs. GG1; *APC P* = 0.009, *GSTP1 P* = 0.004, *RARβ2 P* = 0.008) (Fig. 2a), and the same was observed for *miR-34b/c* (*P* = 0.006) (Fig. 2b). Moreover, increased *APC* methylation levels significantly associated with higher CAPRA Score (*P* = 0.005) (Fig. 2c) and increased risk of recurrence, according with D’Amico’s risk classification (*P* = 0.038) (Fig. 2d). Nevertheless, no association was depicted between promoter methylation levels and patient’s age, diagnostic serum PSA levels, or clinical stage.

In cohort #2, no association between methylation levels and prognostic grade group, pathological stage, or diagnostic serum PSA levels was found. Nonetheless, *miR-34b/c* promoter methylation levels significantly associated (although modestly) with patients’ age at diagnosis (*P* = 0.018, *r* = 0.253). Thus, ROC curves were normalized for this variable and diagnostic performance was calculated accordingly.

### Prognostic value of panel #1 and panel #2 in cohort #1

Prognostic value was only tested in the cohort of PCa patients with longer follow-up (cohort #1), which displayed a median follow-up of 104.04 (range: 67.03–145.48) months. From the 74 patients, only 39 (52.70%) were treated curative intent. All patients were included in DSS analysis (*n* = 74), whereas for DFS, only patients with initial curative intention (RP/RT) but one (without remission) were included (*n* = 38). For PFS analysis, 3 patients were excluded from cohort #1 due to persistence of high serum PSA levels after treatment. During this period, 13/74 (17.57%) patients deceased from PCa, while disease progressed in 29/71 (40.85%). Moreover, 13/38 (34.21%) of patients with initial curative intention developed biochemical recurrence.

**Fig. 3:**
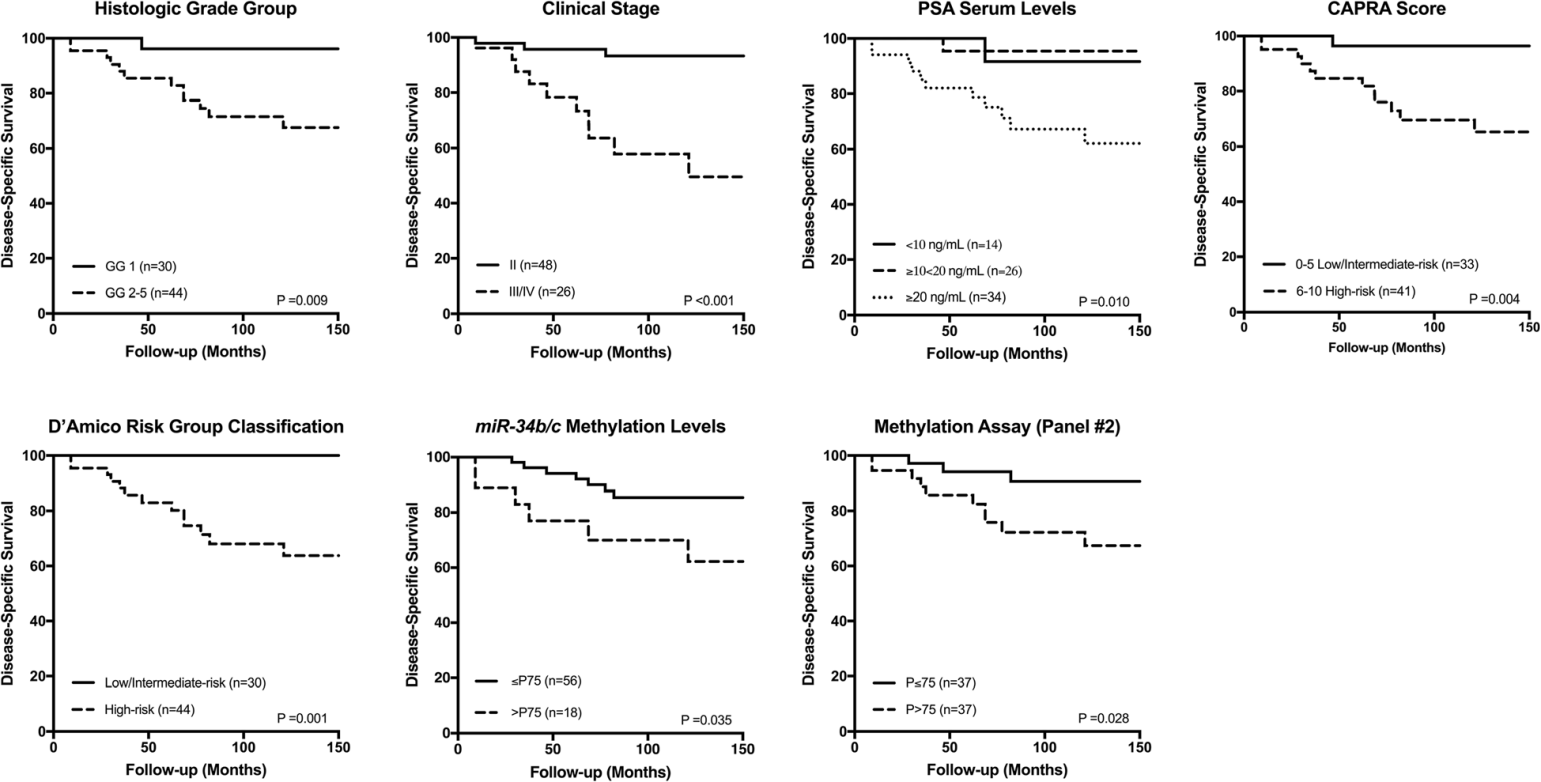
Disease-specific survival curves according to standard clinicopathological parameters [histologic grade group, clinical stage, PSA serum levels, CAPRA Score, and D’Amico risk group classification] and epigenetic marks [*miR-34b/c* and panel #2] in prostate biopsy (cohort #1)

**Fig. 4:**
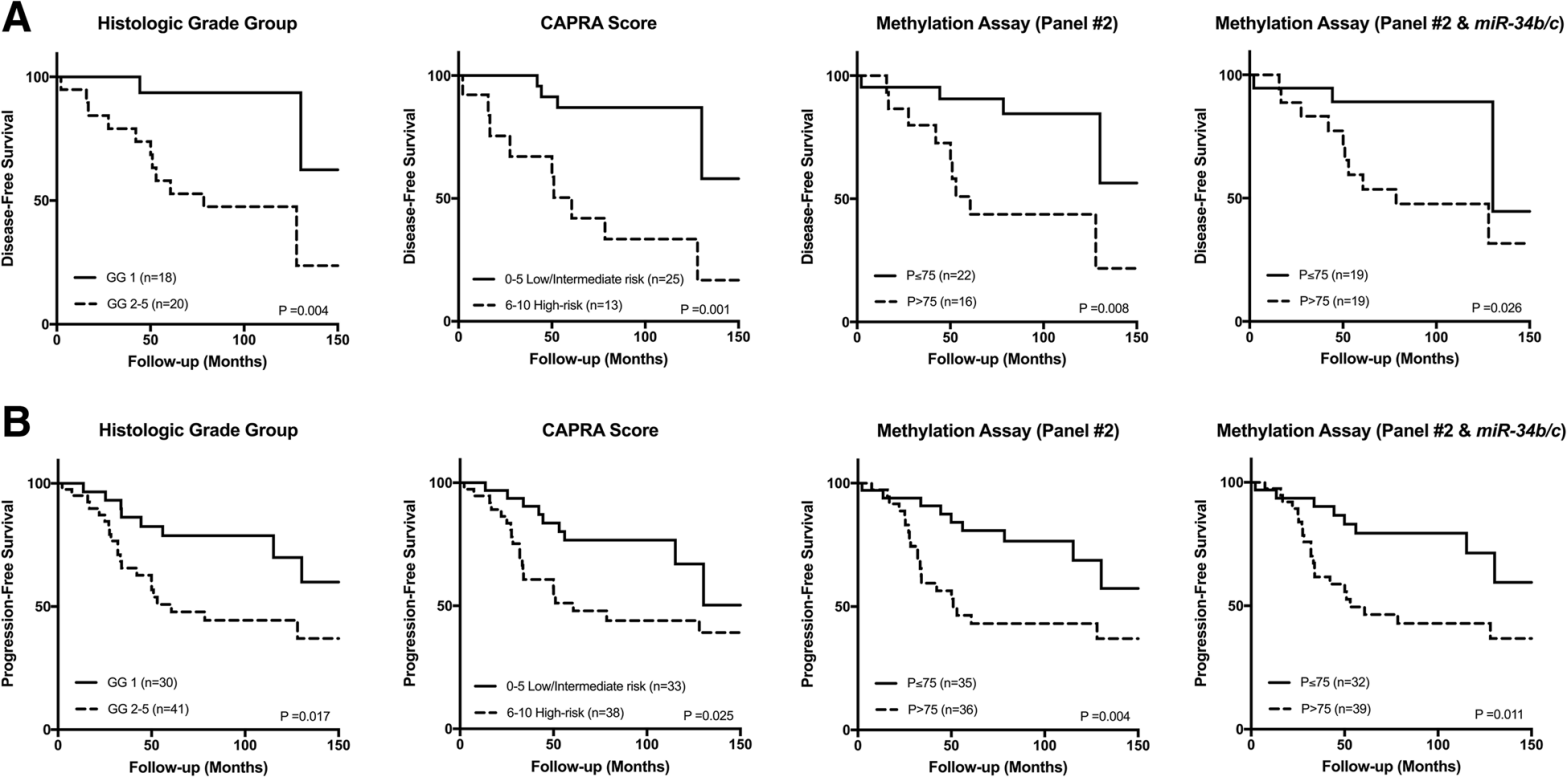
Disease-free survival and progression-free survival curves in prostate biopsy (cohort #1). **a** Disease-free survival curves according to standard clinicopathological parameters [prognostic grade group and CAPRA Score], panel #2 and panel #2 plus *miR-34b/c* promoter methylation levels **b** Progression-free survival curves according to standard clinicopathological features [prognostic grade group and CAPRA Score] and panel #2 and panel #2 plus *miR-34b/c* promoter methylation levels

Regarding DSS, higher prognostic grade group (*P* = 0.009), advanced clinical stage (*P* < 0.001), high diagnostic serum PSA levels (*P* = 0.010), increased CAPRA score (*P* = 0.004), and D’Amico risk classification (*P* = 0.001) significantly associated with shorter survival. A similar result was found for higher *miR-34b/c* (*P* = 0.035) and panel #2 (*APC*, *GSTP1*, and *RARβ2*) (*P* = 0.028) promoter methylation levels (Fig. 3). Furthermore, in multivariable Cox-regression analysis, clinical stage (II vs. III/IV) and *miR-34b/c* or panel #2 methylation independently predicted DSS (Table 5). On the other hand, high prognostic grade group (*P* = 0.004), increased CAPRA score (*P* = 0.001) and higher panel #2 methylation levels (*P* = 0.008) significantly associated with biochemical relapse, but only in univariable analysis. Although panel #1 methylation levels did not associate with DFS, when *miR-34b/c* promoter methylation levels were added to panel #2, higher promoter methylation of this combination predicted shorter time to relapse (*P* = 0.026) (Fig. 4a). Indeed, the combination of *miR-34b/c* (panel #1) and panel #2 methylation levels also associated with shorter PFS in univariable analysis (*P* = 0.011), as well as clinical parameters [high prognostic grade group (*P* = 0.017) and increased CAPRA score (*P* = 0.025)] (Fig. 4b).

**Table 3:** Diagnostic performance of DNA methylation-based biomarkers in cohort #1

Parameters	Panel #1	Panel #2
Sensitivity %	97.3	100.0
Specificity %	80.0	100.0
Positive predictive value %	96.0	100.0
Negative predictive value %	85.7	100.0
Accuracy %	94.4	100.0
Positive likelihood ratio (LR+)	4.87	–
Negative likelihood ratio (LR−)	0.03	–

**Table 4:** Diagnostic performance of DNA methylation-based biomarkers in cohort #2

Parameters	Panel #1	Panel #2	Panels #1 and #2
Sensitivity %	95.4	94.3	100.0
Specificity %	84.4	84.4	75.0
Positive predictive value %	94.3	94.3	91.6
Negative predictive value %	87.1	84.4	100.0
Accuracy %	92.4	91.6	93.3
Positive likelihood ratio (LR+)	6.12	6.04	4.00
Negative likelihood ratio (LR−)	0.05	0.07	-

**Table 5:** Cox-regression models assessing the potential of clinical and epigenetics variables in the prediction of disease-specific survival in cohort #1

Disease-specific survival	Variable	Hazard ratio (HR)	95% CI for OR	*P* value
Multivariable	Clinical stage			
	III/IV	9.637	2.597–35.763	0.001
	Panel #1: *miR-34b/c*			
	Promoter methylation > P75	3.843	1.268–11.649	0.017
	Clinical stage			
	III/IV	8.334	2.274–30.548	0.001
	Panel #2			
	Promoter methylation > P75	3.786	1.038–13.810	0.044

*HR* hazard ratio

**Fig. 5:**
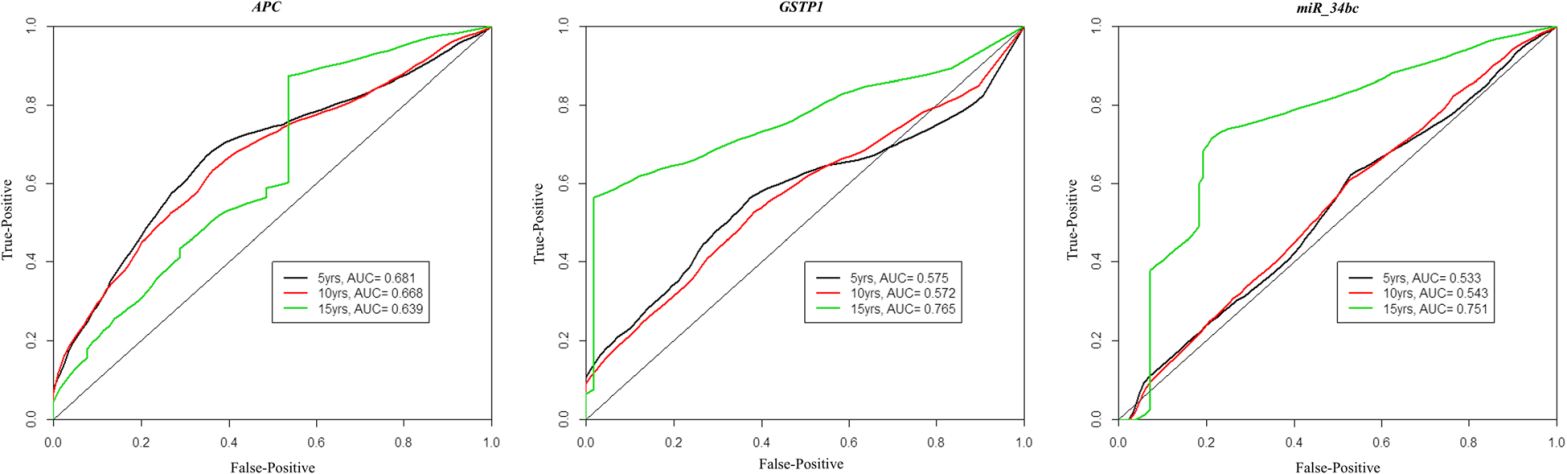
Time-dependent ROC curves at 5, 10, and 15 years follow-up time having as endpoint biochemical recurrence/progression of disease for *APC*, *GSTP1*, and *miR-34b/c* in cohort #1

Furthermore, the prognostic performance of the methylation panels over time was analyzed by constructing time-dependent ROC curves (Fig. 5). Among panel #1 markers, *miR-34b/c* methylation levels showed the best performance at 15 years of follow-up (AUC = 0.751), whereas no significant information was provided by *miR-193b* methylation levels. Concerning panel #2, *APC* and *GSTP1* methylation levels displayed the best performance at 5 years (AUC = 0.681) and at 15 years (AUC = 0.765), respectively, whereas no significant information was provided by *RARβ2* methylation levels.

## Discussion

Prostate cancer is the second most incident solid malignancy in men, being associated with significant morbidity and mortality (5th leading cause of cancer-related death) [20]. The implementation of serum PSA-based screening resulted in an increased number of men diagnosed with PCa at asymptomatic, early stage disease, but this was achieved at the cost of overdiagnosis and overtreatment of clinically insignificant disease. Indeed, serum PSA testing does not accurately discriminate between benign and malignant prostate disease, neither between indolent and clinically significant PCa [21]. Thus, the identification of robust and minimally invasive molecular biomarkers that may be used for screening, diagnosis, risk stratification, and prognostication is mandatory [22, 23]. Owing to the prevalence and cancer specificity of aberrant promoter methylation of both protein-coding and non-coding genes in PCa [24, 25], these constitute promising biomarkers for clinical use [11]. Herein, we compared the biomarker performance of two methylation panels [panel #1 (*miR-193b*/*miR-34b/c*) and panel #2 (*APC*/*GSTP1*/*RARβ2*)], for PCa diagnosis and prognostication, in the same series of prostate biopsies and urine sediments, from two independent cohorts.

In prostate biopsy samples, both panels demonstrated excellent performance for PCa detection, although panel #2 attained maximal performance, emphasizing the potential of these panels as ancillary tools for PCa detection in this setting. These results are in line with three previously reported multicenter studies, in which *APC*/*GSTP1*/*RASSF1A* multiplex quantitative methylation analysis in biopsy needle core tissue samples with histological negative result displayed 88–100% NPV, confirming methylation assays as helpful tools for assisting in decision of re-biopsy [26–28]. Indeed, besides the need to precisely identify PCa in biopsy, accurately deny of its presence is of paramount importance as this will allow for extending the time period until re-biopsy, significantly reducing costs and harmful side-effects of an invasive procedure. However, because only biopsy cores containing PCa were included and not negative cores, nor the respective prostatectomy specimens, this maximal sensitivity might not be confirmed definitively. Panel #1, however, displayed 97.3% sensitivity. This value obtained in the same sample set clearly indicates that although the vast majority of PCa cases display miR-34b/c and/or miR-193b promoter hypermethylation, 2.7% of cases do not, which might be due to tumor heterogeneity.

Notwithstanding the potential of methylation-based biomarkers in tissue samples, its use for early detection requires assessment in body fluids. In this setting, panel #1 identified PCa with higher sensitivity than panel #2, but with modest specificity, providing an AUC of 0.8836, in urine sediments. Unexpectedly, the panel #1 AUC was lower than previously reported by us [11] which might be explained by differences in the control population. On the other hand, the methylation panel comprising *APC* and *RARβ2* identified PCa with only fair sensitivity and specificity, although with higher AUC compared to panel #1. The inclusion of only two genes (*APC* and *RARβ2*) was due to the lack of significant differences of *GSTP1* promoter methylation levels between PCa urine samples and controls. This was a rather unexpected finding considering previous reports from our group and others [14, 29–31], and it might be due to different procedures for urine collection. Indeed, the urine samples used in this study were not collected after DRE or prostatic massage, which increase shedding of prostate cells, thus improving sensitivity [32]. Nevertheless, the two-gene panel (*APC* and *RARβ2*), displayed better sensitivity and AUC than the ProCaM assay, which is a three-gene panel [10]. Moreover, in that study, promoter methylation analysis was performed in a two-step PCR, whereas we carried out a one-step PCR, allowing for faster and more cost-effective detection.

Remarkably, the combination of the two methylation panels depicted maximal sensitivity and NPV, improving accuracy (93.3%) and AUC (0.9817), thus augmenting PCa detection performance in urine. Also, the methylation test encompassing the two panels present null LR—, indicating that there is no chance of disease when the test is negative, and a LR+ of 4. As previously mentioned, the accuracy of serum PSA testing to predict PCa is suboptimal [21] and regardless the efforts to improve its performance, namely through PSA-derived parameters, the added value is small [33]. On the contrary, our results show that a methylation assay combining panel #1 and panel #2 displays a diagnostic performance in urine sediments superior to serum PSA and urinary PCA3, which are currently used in clinical practice [34]. Nevertheless, future studies that include larger cohorts of PCa patients and healthy subjects are required to further validate these preliminary results and provide direct comparisons among the several tests.

Because overdiagnosis and consequent overtreatment are a major concern, PCa detection should be accompanied by risk stratification for disease aggressiveness. Thus, the ability to convey relevant prognostic information is of chief importance. Interestingly, at the time of diagnosis, higher promoter methylation of *miR-34b/c* and panel #2 genes associated with clinicopathological features of disease aggressiveness (prognostic grade group, CAPRA score, D’Amico’s risk classification), suggesting the ability to discriminate clinically significant from indolent PCa, in biopsy samples, probably due to the increased promoter methylation of several target genes along PCa initiation and progression. Notwithstanding these correlates, survival analysis is critical to assess the prognostic significance. Remarkably, *miR-34b/*c from panel #1 and panel #2 promoter methylations disclosed independent prognostic significance, along with clinical stage, suggesting a role as stratification tool for disease aggressiveness, and in line with a previous study from our group [11, 13]. Furthermore, we found that the prognostic ability of methylation biomarkers is time dependent. Indeed, both *miR-34b/c* and *GSTP1* promoter methylations increase its prediction accuracy over time, identifying patients that relapse up to 15 years after diagnosis and beyond. Thus, promoter methylation of these genes might allow for more personalized follow-up procedures, increasing the cost-effectiveness of PCa patients’ management in the long term.

The main limitation of this study is the relatively small sample size, in both cohorts, that might preclude the identification of more significant differences between promoter methylation levels in tissue and urine samples and patient outcome. Furthermore, follow-up time of cohort #2 does not allow for the assessment of the prognostic value of methylation biomarkers in urine. Nevertheless, it should be emphasized that all samples were prospectively collected and that a multiplex assay was used, allowing for diagnostic and prognostic assessment of PCa suspects in a single analysis as a mean to improve the efficiency of the test, considering the limited amount of prostate cell-derived DNA present in urine. Importantly, although our results suggest a potential clinical usefulness, prospective validation (which we did not perform) in an independent cohort is mandatory.

## Conclusions

**Fig. 6:**
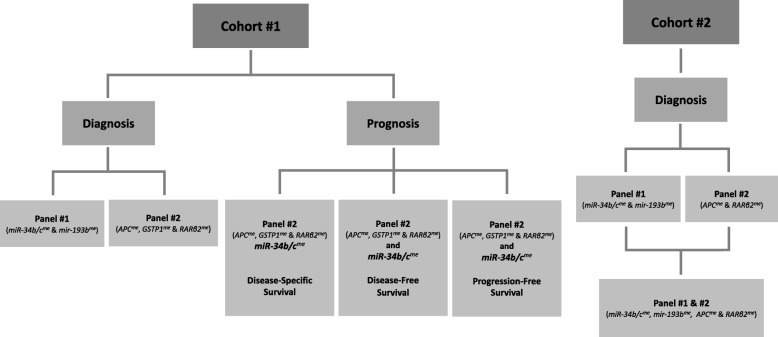
Flow chart of tested diagnostic and prognostic biomarkers in the two studied cohorts of patients

In conclusion, this study highlights the potential of specific gene promoter methylation testing in biopsy and urine sediments for PCa detection and prognostication (Fig. 6). Furthermore, the multiplex methylation assay of panel #2 constitutes a powerful method for methylation analysis of samples with minimal DNA amounts. Nonetheless, the potential usefulness of these DNA methylation biomarkers requires further studies in larger series of PCa suspects, to develop a valid tool for accurate detection of clinically significant PCa.

## Additional files

Additional file 1: **Table S1.** Primer sequences for *β-Actin*, *miR-34b/c*, and *miR-193b* (panel #1). Table S2. Scorpion primer-probe and primer inner sequences for *β-Actin*, *APC*, *GSTP1*, and *RARβ2* (panel #2). Table S3. Diagnostic performance of each DNA methylation-based biomarker in cohort #1. Table S4. Diagnostic performance of each DNA methylation-based biomarker in cohort #2. (DOCX 22 kb)

Additional file 2: **Figure S1.** Box-plots of panel #1 (*miR-34b/c*, *miR-193b*) and panel #2 (*APC*, *GSTP1*, and *RARβ2*) promoter methylation levels in morphologically normal prostatic tissue (MNPT) and prostate cancer patients submitted to prostate biopsy (PCa) (cohort #1). (Mann-Whitney *U* test, ****P < 0.0001). (TIFF 1344 kb)

Additional file 3: **Figure S2.** Box-plots of panel #1 (*miR-34b/c*, *miR-193b*) and panel #2 (*APC*, *RARβ2*) promoter methylation levels in asymptomatic controls (AC) and in prostate cancer (PCa) urine samples (cohort #2). (Mann-Whitney *U* test, ****P < 0.0001). (TIFF 1410 kb)

## Abbreviations

AC: Asymptomatic controls
APC: Adenomatosis polyposis coli
AUC: Area under curve
CAPRA score: Cancer of the prostate risk assessment score
DFS: Disease-free survival
DSS: Disease-specific survival
GSTP1: Glutathione S-transferase pi 1
IQR: Interquartile range
LR+/−: Positive/negative likelihood ratio
miR: MicroRNA
MNTP: Morphologically normal prostate tissue
NPV: Negative predictive value
PCa: Prostate cancer
PCR: Polymerase chain reaction
PFS: Progression-free survival
PPV: Positive predictive value
ProCAM: Prostate cancer methylation
PSA: Prostate-specific antigen
RARβ2: Retinoic acid receptor β 2
