# Supplementary material for: Comparing diagnostic and prognostic performance of two-gene promoter methylation panels in tissue biopsies and urines of prostate cancer patients
Source: Clin Epigenetics. 2018 Oct 29;10:132. doi: 10.1186/s13148-018-0564-2 (PMC6206889; doi:10.1186/s13148-018-0564-2)
Supplement: Supplementary file 1 — Table S1. Primer sequences for β-Actin, miR-34b/c, and miR-193b (panel #1). Table S2. Scorpion primer-probe and primer inner sequences for β-Actin, APC, GSTP1, and RARβ2 (panel #2). Table S3. Diagnostic performance of each DNA methylation-based biomarker in cohort #1. Table S4. Diagnostic performance of each DNA methylation-based biomarker in cohort #2. (DOCX 22 kb) [file 13148_2018_564_MOESM1_ESM.docx]

**Comparing diagnostic and prognostic performance of two gene promoter methylation panels in tissue biopsies and urines of prostate cancer patients**

**Moreira-Barbosa et al**

**Table S1 Primer sequences for *ß-Actin*, *miR-34b/c* and *miR-193b* (Panel #1).**

| **Gene** | **Forward** | **Reverse** |
| --- | --- | --- |
| ***ß-Actin*** | TGGTGATGGAGGAGGTTTAGTAAGT | AACCAATAAAACCTACTCCTCCCTTAA |
| ***miR-34b/c*** | GTTTAGTTACGCGTGTTGTGC | CGAAAAACGCCCTACCATA |
| ***miR-193b*** | AGCGGGGTGTTTGTGTTC | AAACATAAACGACGCATTCCA |

**Table S2 Scorpion primer-probe and primer inner sequences for *ß-Actin*, *APC*, *GSTP1* and *RARß2* (Panel #2).**

| Gene | Primer | Sequence (5´-3´) | Reference |
| --- | --- | --- | --- |
| *β-Actin* | Scorpion probe-primer | [Cyanine5]CCGGGGCCTCCATCACCACCCCGG[BHQ2dT][HEG]TATAGGTTGGGGAAGTTTGTTTTTG | [15] |
|  | Inner Primer | AACACACAATAACAAACACAAATTCAC |  |
| *APC* | Scorpion probe-primer | [6FAM]CCGCGCTATGTGGTTGTATTGGTGTAGTTCGTGCGCGG[BHQ2dT][HEG]CGACAACACCTCCATTCTATCTCCAATA | - |
|  | Inner Primer | TTGGTTGGGTGTGGGCGTAC |  |
| *GSTP1* | Scorpion probe-primer | [Cyanine3]CCGGGCGAACTCCCGCCGAGCCCGG[BHQ2dT][HEG]TCGGGGTGTAGCGGTCGTCG | [15] |
|  | Inner Primer | CGCCCCAATACTAAATCACG |  |
| *RARβ2* | Scorpion probe-primer | [JOE]CGGCGCCCGACGATACCCAAAGCGCCG[BHQ1dT][HEG]AACGCGAGCGATTCGAGTAG | [14] |
|  | Inner Primer | CTTACAAAAAACCTTCCGAATACG |  |

**Table S3 Diagnostic performance of each DNA methylation-based biomarker in Cohort #1**

| Cohort #1 | Panel #1 | | Panel #2 | | |
| --- | --- | --- | --- | --- | --- |
|  | *miR-34b/c* | *miR-193b* | *APC* | *GSTP1* | *RARß2* |
| Cut-off | 104.04 | 81.89 | 25.31 | 67.59 | 92.71 |
| Sensitivity % | 87.8 | 81.1 | 100 | 83.8 | 89.2 |
| Specificity % | 80.0 | 100 | 100 | 100 | 100 |
| Positive predictive value % | 95.6 | 100 | 100 | 100 | 100 |
| Negative predictive value % | 57.1 | 51.7 | 100 | 55.6 | 65.2 |
| Accuracy % | 86.5 | 84.3 | 100 | 86.5 | 91.0 |
| Positive likelihood ratio (LR+) | 4.39 | - | - | - | - |
| Negative likelihood ratio (LR-) | 0.15 | 0.19 | - | 0.16 | 0.11 |

**Table S4 Diagnostic performance of each DNA methylation-based biomarker in Cohort #2**

| Cohort #2 | Panel #1 | | Panel #2 | |
| --- | --- | --- | --- | --- |
|  | *miR-34b/c* | *miR-193b* | *APC* | *RARß2* |
| Cut-off | 0.79 | 34.00 | 21.35 | 0.66 |
| Sensitivity % | 71.3 | 93.1 | 88.5 | 44.8 |
| Specificity % | 96.9 | 84.4 | 87.5 | 96.9 |
| Positive predictive value % | 98.4 | 94.2 | 95.1 | 97.5 |
| Negative predictive value % | 55.4 | 81.8 | 73.7 | 39.2 |
| Accuracy % | 78.2 | 90.8 | 88.2 | 58.8 |
| Positive likelihood ratio (LR+) | 22.80 | 5.96 | 7.08 | 14.34 |
| Negative likelihood ratio (LR-) | 0.30 | 0.08 | 0.13 | 0.57 |
